# Morphology and Applications of Self-Assembled Peptide Nucleic Acids

**DOI:** 10.3390/ijms252212435

**Published:** 2024-11-19

**Authors:** Luca Domenico D’Andrea, Alessandra Romanelli

**Affiliations:** 1 Istituto di Scienze e Tecnologie Chimiche “Giulio Natta”, Consiglio Nazionale delle Ricerche, via M. Bianco 9, 20131 Milano, Italy; luca.dandrea@cnr.it; 2 Dipartimento di Scienze Farmaceutiche, Università degli Studi di Milano, via G. Venezian 21, 20133 Milan, Italy

**Keywords:** peptide nucleic acid, assembly, fiber, micelle, hydrogel

## Abstract

Obtaining new materials by exploiting the self-assembly of biomolecules is a very challenging field. In recent years, short peptides and nucleic acids have been used as scaffolds to produce supramolecular structures for different applications in the biomedical and technological fields. In this review, we will focus on the self-assembly of peptide nucleic acids (PNAs), their conjugates with peptides, or other molecules. We will describe the physical properties of the assembled systems and, where described, the application they were designed for.

## 1. Introduction

Biomolecules such as peptides and nucleic acids can form supramolecular structures in which the single units are held together by non-covalent interactions. In nucleic acids, the recognition of complementary bases allows for the construction of 1D to 3D supramolecular structures, upon a proper choice of the oligonucleotide sequence [1,2]. In peptides, hydrogen bonds between the backbone and interactions involving the amino acid side chain may trigger the formation of supramolecular systems [3,4].

The combination of peptides or proteins and DNA in a single molecule results in hybrid nanostructures that find application in many areas of nanotechnology [5,6]. These assemblies are biocompatible and biodegradable and are employed in different fields, for example, as delivery systems, in tissue engineering applications, or as sensors or catalysts [7,8,9]. A considerable effort is devoted to the development of scaffolds that assemble in a predictable way to give ordered structures, endowed with specific functions.

In recent years, there has been a growing interest in the self-assembly of nucleic acid analogs such as peptide nucleic acids (PNAs).

In these molecules, nucleobases are attached to a pseudopeptide backbone; the sequence-specific recognition of complementary nucleobases occurs by Watson–Crick or Hoogsteen hydrogen bonds [10]. The PNA backbone is neutral; this feature results in a lack of electrostatic repulsion between complementary strands and therefore in a more marked tendency to aggregate as compared to DNA or RNA. Unlike DNA, which can be obtained either by chemical synthesis or enzymatic reactions, PNAs can only be produced by chemical synthesis. Robust protocols are now available for the solid-phase synthesis of standard PNAs and PNAs modified on the backbone by amino acid side chains or other organic molecules [11,12,13,14]. The variety of functional groups that can be appended to PNAs allows for the application of these molecules in various fields [15]. Several papers have been focused on the application of PNAs targeting DNA or RNA, in applications such as antisense, antigene, antagomir, or biosensors [16,17,18]. The in vivo use of these molecules is limited by their low bioavailability and the limited tools optimized to promote their cellular uptake [19,20]. Recently, the possibility of exploiting PNAs to produce supramolecular aggregates has been explored [21]. PNAs can self-assemble by exploiting interactions between the modules of which it is composed, i.e., nucleobases, the peptide backbone, and the amino acid side chains present in PNA analogs; thus, in principle, it is possible to produce different structures combining the modules in different ways. In many cases, PNAs have been conjugated to self-assembling scaffolds with the aim of improving the physical or mechanical properties of the aggregates. 

**Figure 1:**
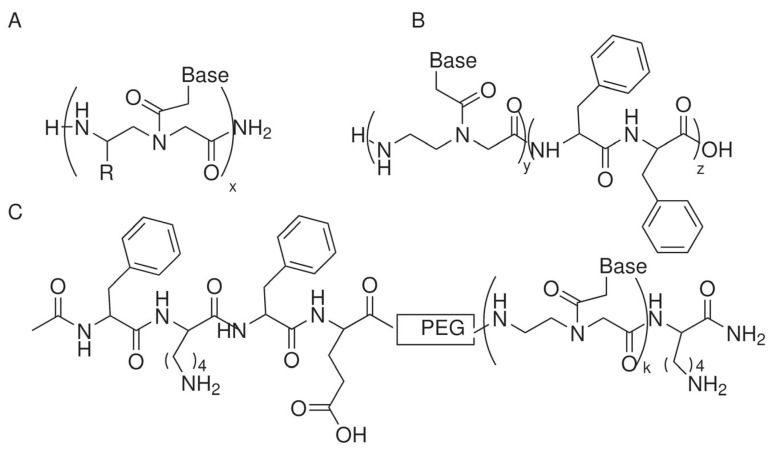
Chemical structure of selected PNAs assembling to produce fibers: (**A**) R = H, X = 2 [22]; R= H or CH_2_OH, x= 12 [23]; (**B**) y = 1,2 and z = 1,2 [24,25]; (**C**) k = 10 [26].

**Figure 2:**
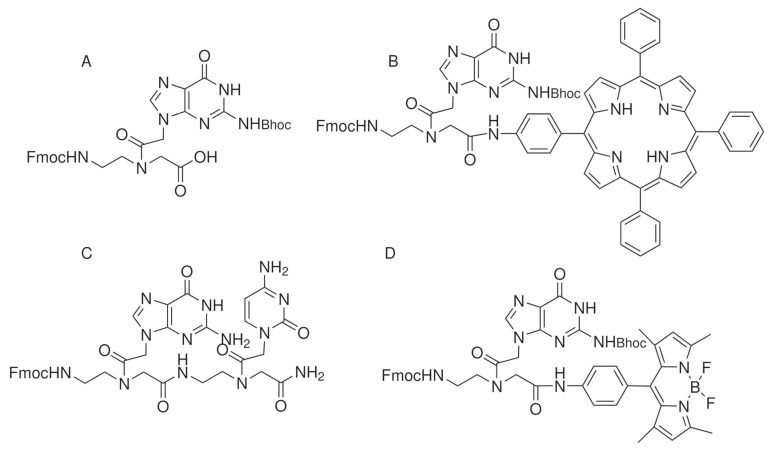
Chemical structure of selected PNAs assembling to produce spheres: (**A**) [27]; (**B**,**D**) [28]; (**C**) [29].

**Figure 3:**
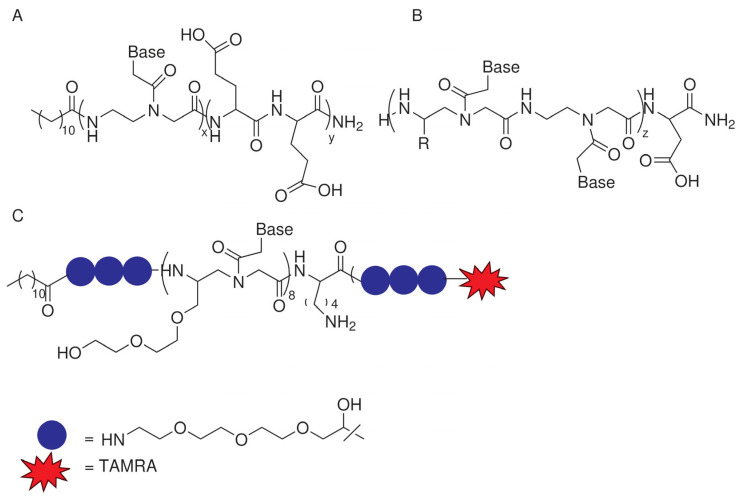
Chemical structure of selected PNAs assembling to produce micelles: (**A**) [30]; (**B**) [31,32]; (**C**) [33].

The first studies reporting the ability of PNAs to self-assemble were focused on long PNA oligomers. Later, short PNAs were investigated, and the effects of different modifications to the backbone or the conjugation to peptides or alkyl chains on the self-assembling process were reported. In this review, we will discuss the assembly conditions, properties, and applications of self-assembled PNAs and selected systems in which the supramolecular system contains molecules other than PNAs such as nucleic acids, peptides, or small molecules. A wide variety of structures can be obtained by changing the composition of the molecule (Table 1). Figure 1, Figure 2 and Figure 3 show the chemical structures of the selected molecules able to assemble to produce fibers, spheres, or micelles. 

## 2. Discussion

### 2.1. Long PNA Oligomers

In 2003, Cao et al. reported the first example of a hydrogel containing a PNA sequence [34]. Microgels were obtained by exploiting a three-component system, containing three hybridized DNA oligomers that form a three-way junction (TWJ), biotinylated PNAs complementary to the single-strand regions of the TWJ, and streptavidin. The high affinity of PNAs toward complementary DNA is exploited to build the supramolecular structure. The PNAs crosslink the TWJ to the protein. Gelation occurs at pH 7, after the hybridization of PNA and DNA strands. Microgels are thermoreversible, the gelation temperature is dependent on the length and sequence of the PNA/DNA duplex. The non-covalent crosslinking of fibrils due to the recognition between complementary PNAs and DNA results in the rigidification of the hydrogel.

Using a strategy similar to that reported by Cao, PNA/DNA hybrids were used to connect polymeric fibers to obtain a hydrogel. In a previous study, 8- or 11-mer PNA oligomers were conjugated to N-methacryloylglycylglycine at the N-terminus to produce copolymers with N-(2-hydroxypropyl)methacrylamide (HPMA) [35]. PolyHPMA is a hydrophilic and biocompatible polymer, very well characterized in terms of toxicity to the human body. Polymers containing HPMA with PNA grafts were mixed with DNA complementary to the PNAs in water. The DNA sequences were designed to form duplex or triplex hybrids, with the PNAs grafted on the polymers. Hydrogels formed at a critical concentration of 3 wt% polymer–PNAs when they contained PNA/DNA duplexes. Instead, when triple helices were formed, the critical gelling concentration was lower. SEM analyses revealed that the hydrogels possess a porous structure, with larger pore size in the hydrogel formed using PNA/DNA duplexes. The stiffness of the hydrogel is related to the crosslinking density, i.e., to the number of grafted PNAs. 

In 2010, Becker et al. [36] reported the formation of hollow capsules obtained by the sequence-directed layer-by-layer deposition of 16-mer PNA oligomers (Glu-tgagcttg tatagtgc- and Glu-caagctca cgactctc-Glu) on amine-functionalized silica nanoparticles. The PNAs contain equal amounts of purine and pyrimidines; each oligomer contains two blocks designed to be complementary to the blocks on the other PNA. The C- and N-terminal ends are linked to a glutamic acid to improve solubility. Upon the removal of the core, a stable capsule formed. The application of these capsules for delivery was proposed.

Self-assembly induced by the addition of small molecules was described by Avakyan in 2016. The formation of fibers was reported for polyadenine PNA oligomers (a_7_) in the presence of cyanuric acid (CA) [37]. CA units possess three thymine-like faces; each CA binds two different adenine nucleobases, and each adenine exploits both Watson–Crick and Hoogsteen hydrogen bonds to bind two CA units. CA and adenine organize into a hexameric rosette structure; polyadenines polymerize in a cooperative fashion, forming triple helices. AFM images show fibers with an average length of 100 nm and a diameter of 2.0 nm.

### 2.2. PNA Monomers 

PNA monomers do not self-assemble, as demonstrated by Gazit in a comprehensive study in which different buffers, organic solvents, and monomer concentrations were investigated [22]. Masking the exocyclic amine on the nucleobase and/or the primary amine on the backbone with protecting groups or organic, hydrophobic moieties results in molecules with increased hydrophobicity and marked ability to produce supramolecular structures. Studies on PNA monomers are mostly focused on guanine. This nucleobase, in fact, can form either Watson–Crick and Hoogsteen hydrogen bonds and can produce G-quartets (observed in G-quadruplex structures). Guanine forms supramolecular structures with interesting optical properties; these are referred to as photonic crystals, i.e., crystals able to change color upon an external stimulus [38]. These structures are found on the skin of some animals able to change the color of their skin as chameleons. 

In terms of the assembly of fully protected guanine monomers, Fmoc-g(Bhoc)-OH has been widely investigated (Figure 2A). In 2016, Berger reported that upon dissolution in water at high temperatures and at a concentration of 5 mg/mL, it assembles into spheres; at the air–water interface, a small fraction of spheres further assemble into a thin layer, exhibiting a purple color [27]. Upon the addition of NaCl, crystals further change color. The organization of the spheres of PNAs is similar to the organization of guanine on the skin of chameleons. Change in the color of the skin is due to a modification of the geometry of the photonic crystals, resulting in a change in the wavelength of the refracted light. In the PNA spheres, changes observed following the addition of NaCl are due to a reduction in the electrostatic potential that surrounds spheres in solution.

In a more recent paper, Zhang et al. reported that drop-casting a Fmoc-g(Bhoc)-OH solution at 2 mg/mL concentration in water at different temperatures produces adaptive photonic crystals (PCs) [39]. PCs microfabricated at different temperatures displayed different structural colors. The different arrangement of microspheres was confirmed by XRD analysis. SEM showed regular microspheres packed in a hexagonal arrangement when the assembly was carried out at 20 and 40 °C, while irregular aggregates were formed at higher temperatures. PCs exhibit solvent-polarity-dependent solvatochromism and may be employed for the detection of organic solvents of different polarities.

The self-assembling ability of fully protected guanine monomers was exploited to produce supramolecular catalytic systems, upon conjugation to the monomer of proper organic moieties.

Fmoc-g(Bhoc) PNA monomer was conjugated to chromophores such as porphyrin (TPP) and bodipy (BDP) (Figure 2B,D). The conjugates formed supramolecular aggregates with different morphologies in organic solvents at concentrations between 1 and 7 mM [28]. PNA-TPP formed nanospheres, while PNA-BDP formed spherical or flake-shaped aggregates depending on the solvent employed. Fluorescence experiments demonstrated that an energy or electron transfer process from the PNA monomer to the chromophore occurred even when the conjugates were in their assembled state. The ability of the conjugates to harvest light was exploited in the photocatalytic evolution of H_2_ in an aqueous environment in the presence of Pt nanoparticles. The results obtained successfully demonstrate that nanoassemblies obtained are promising candidates for photocatalytic applications.

The aggregation of PNA-BDP and PNA-TPP was also investigated in aqueous conditions [40]. The formation of spherical nanoparticles with an average diameter of 100 nm was observed. The nanoparticles exhibited stability upon dilution as well as in cell media and were internalized by cells without showing cytotoxicity in the dark. Importantly, upon irradiation, cells were efficiently killed by the nanoparticles, thanks to the ability of the BDP or TPP to act as photosensitizers. The application of these systems in photodynamic therapy can be envisaged.

The introduction of alkyl chains at the C-terminus of Fmoc–guanine affects the supramolecular organization of guanine. Fmoc-g conjugated to aminopentanoic acid at the C-terminus self-assembles in different morphologies from methanol/water solutions, at different concentrations [41]. At low concentrations of up to 0.1 mg/mL, spheres are formed, while at higher concentrations (0.5 mg/mL or higher) nanoribbon assemblies are formed. In water at 0.1 mg/mL, upon heating at 100 °C and after filtering, microspheres with a rough surface and distributed micropores were observed. Guanines arrange to form tetramers, stabilized by head-to-tail packing, in an arrangement that recalls G-quadruplexes, which stack one on top of the other. The Young modulus of the crystal was found to be significantly higher than that measured for self-assembled DNA nanostructures. The G-quartets exhibited fluorescence emission with a longer lifetime and higher quantum yield compared to assemblies formed by Fmoc-gc [29].

The PNA G-quartet was employed to build an artificial photosynthetic system comprising a ruthenium-based light-harvesting antenna and a platinum catalytic center [42]. PNA crystals were dispersed in a solution of the ruthenium complex, resulting in the deposition of the metal complex on the PNA structure. The PNA/Ru crystals were demonstrated to be able to produce photoelectrons and show electro-redox activity. Pt nanoparticles were integrated into the PNA/Ru crystal by electron beam evaporation. The light-driven conversion of NAD^+^ to NADH was investigated in the presence of the electron donor triethanolamine. NADH generation occurred with an efficiency comparable to that observed with self-assembled peptides, demonstrating the efficiency of PNA crystals in energy transfer processes.

### 2.3. PNA Dimers

Pioneer studies on the ability of short PNA sequences to aggregate were reported by Gazit’s group in 2015 (Figure 2A) [22]. The ability of PNA monomers and dimers in all possible combinations, in different organic solvents, and in aqueous conditions at different pH levels was investigated. Only dimers containing guanine such as gc, cg, and gg could self-assemble into ordered structures at pH 11, endowed with the ability to emit fluorescence in a wide range of wavelengths. In these assembled systems, upon increasing the excitation wavelength, an increase in the emission wavelength was observed. This phenomenon is called REES (red-edge excitation shift). The X-ray crystal structure of the assembled gc revealed that Watson–Crick hydrogen bonds and π-π stacking hold the dimer together. The ability of assembled gc to be used in optoelectronic as organic light-emitting materials was successfully demonstrated.

The coassembly of PNA dimers gg and cc in bicine buffer at pH 9 using a microfluidic system produces well-ordered structures, stabilized by Watson–Crick hydrogen bonds [43]. As observed for gc aggregates, these also display REES, i.e., a shift in the emission peak to the red edge of the electromagnetic spectrum after increasing the excitation wavelength.

The assembly of gc dimers protected by a Fmoc group at the N-terminus was reported by Avitabile et al. in 2018 (Figure 2C) [29]. The presence of the Fmoc group renders this molecule soluble in organic solvents. In chloroform/methanol mixtures, Fmoc-gc assembled at 0.5 mg/mL concentration to produce spherical objects. NMR studies revealed that the structure was stabilized by hydrogen bonds between complementary nucleobases and π-π interaction between the fluorenyl group. The aggregate emitted fluorescence in the visible region, as already observed for gc, with high quantum yields and lifetimes in the nanosecond scale.

The assembly of gg conjugated at the N-terminus to a C12 chain (alkyl or alkoxy) was investigated in DMSO/water 2/8. *v*/*v* [44]. At a 0.1 mM concentration, the compound assembled to form spherical structures, like micelles with diameters ranging from 20 to 40 nm, in which guanine was exposed to the solvent. At 1 mM, spheres turned into rod-like structures, which were transformed into fractals upon incubation at 4 °C for 30 days. The appearance of fractals as dendrimers suggested a diffusion-limited aggregation process at the base of fractal formation. The shift in structures upon concentration change was similar to that observed in some PNA–peptide conjugates (WWgc), which will be described later [45].

### 2.4. PNA–Peptide Conjugates

Initial studies refer to PNAs conjugated to charged peptides and alkyl chains to obtain micelles and liposomes by exploiting the hydrophobicity of the hydrocarbon chain and the hydrophilicity of the PNA–peptide moiety. Two more classes of peptide sequences were investigated: hydrophobic peptides, mainly poly-phenylalanines and amphipathic peptides able to form hydrogel. In these cases, peptides play a key role in determining the morphology and properties of the resulting system.

#### 2.4.1. PNA–Peptide–Alkyl Chains

Studies on the aggregation of PNA–peptides conjugated to alkyl chains were reported by Vernille in 2004. PNAs (6 or 10 mer) conjugated to a C12–alkyl chain at the N-terminus and a peptide (KK for a 6-mer PNA or EEEE for a 10-mer PNA) were reported to aggregate at pH 7.0 in phosphate buffer, at a concentration of 15 µM (10 mer) and 40 µM (6 mer). No other characterization of the aggregates was reported (Figure 3A) [30].

More detailed characterization was carried out on PNA amphiphiles obtained by linking a C12–alkyl chain to the PNA–peptide of sequence tagacg-E_2_; this molecule formed ellipsoidal micelles above its CMC of 110 µM at pH 8 (Figure 3B) [31]. Upon the addition of a complementary DNA, micelle formation was prevented. These compounds were designed to be applied in the micellar separation of DNA oligomers.

In a different report, the molecule composed of the C12–alkyl chain conjugated to the sequence ctgactga-E_4_ was described. The PNA sequence was designed so that it self-hybridized in parallel duplexes [46]. At a low concentration (46 µM) in phosphate buffer at pH 7.4, it formed spherical micelles. Micelles were formed if C12 was replaced by C18 or if E_4_ was replaced by E_2_. With an increase in the hydrophilic/hydrophobic ratio, the critical micelle concentration decreased.

PNA amphiphiles containing a PNA sequence (6 to 10 bases) connected through a linker to two alkyl chains (C14) at the N-terminus and a charged peptide (E_4_ or K_2_) at the C-terminus, mixed with cholesterol and DSPC form stable liposomes, which were able to incorporate DNA oligomers complementary to the PNA [47]. 

Finally, PNA–peptide amphiphile composed of a K-t_7_-KK-GGGAAAK(Palm) was shown to form hydrogel at pH above 7 when the concentration was over 1 wt % [48]. Circular dichroism (CD) studies revealed the formation of beta-sheets, and TEM images showed fibers with a diameter of 8 nm and length >100 nm. No PNA signal in the CD spectrum was observed. The formation of gel was reversible when pH was switched to 4.

#### 2.4.2. Hydrophobic Peptide Conjugates

In this section, we report the aggregation properties of short PNAs (monomers or dimers) conjugated to peptides containing up to 5 amino acids.

Datta et al. investigated the self-assembly of the dipeptide diphenylalanine (FF) conjugated through an amide bond or a triazole linker to the four PNA monomers in water/ethanol mixtures [49]. This dipeptide is very well known for its ability to self-assemble and produce nanotubes [50,51]. The effect of N- and C-terminal capping along with the insertion of protecting groups on the nucleobases on the assembly and morphology of the molecules was explored. Unlike fully deprotected molecules, the more hydrophobic conjugates with either end capping or protected nucleobases produced ordered nanoparticles. The adenine conjugates assemble in hollow nanoparticles, are stable in a wide range of pH levels and temperatures, resistant to proteolytic degradation, and can encapsulate carboxyfluorescein. Studies on guanine conjugates reveal their ability to work as supercapacitors.

Diphenylalanine conjugated to PNA monomers and homodimers was reported by Avitabile et al. in 2019 (Figure 1B) [25]. The critical aggregation concentration (CAC) measured in water of molecules composed of dimeric PNAs was lower as compared to that of monomeric PNAs due to π-π stacking. Spectroscopic measurement demonstrated the formation of beta-sheet-like structures that produced fibers, as demonstrated by GIWAXS data and SEM images. These compounds showed fluorescence, as already shown for PNA dimers in solution and the solid state. Investigating the fluorescence of nucleobases in water at high concentrations, the authors demonstrated that the fluorescence of self-assembled PNAs is due to nucleobase aggregation. 

By changing the nucleobase composition, the morphology of the aggregates changes. In studies on FF conjugated to gc heterodimer PNAs, it was reported that these conjugates form disordered spherical-like structures, with spectroscopic features similar to that reported for similar compounds. The relative position of the peptide and the PNA affected the CAC, which is lower when the PNA is at the C-terminal end [24]. The presence of a carboxy vs. an amide at the C-terminal end did not affect aggregation properties. The ability of these molecules to encapsulate nucleic acid was also reported.

When the dipeptide FF was replaced by WW to give WWgc, the molecules maintained their ability to self-assemble and emit fluorescence due to nucleobase aggregation [45]. The morphology of the aggregates was concentration-dependent; spherical structures were observed at pH 5.5 and 4 mg/mL concentration, while intertwined fibers were formed at 10 mg/mL. The structure was stabilized by the interactions between the aromatic amino acids or nucleobases, as suggested by CD spectra and 3D structural models. WWat, unlike WWgc, formed only spherical objects.

Studies on conjugates of tetraphenylalanine to “at” or “gc” PNA dimers (4Fat or 4Fgc) were reported [52]. Aggregation-induced fluorescence was observed for both compounds. CD studies suggested the formation of beta-sheets for 4Fat conjugates that produced long twisted fibers, as suggested by SEM. Notably, 3D structural models suggested the formation of ribbons in which 4Fat building blocks arranged in an antiparallel fashion and nucleobase bases zipped the monomers. When the PNA dimer “at” was replaced by gc, vesicle-like structures were observed.

Molecules composed of alternating phenylalanine (F) and PNA monomers, namely (cFgF)_n_ and (tFaT)_n_, with n = 1 or 2, were recently investigated [53]. Interestingly, the length of the sequences affected the aggregation ability of the compounds; longer sequences exhibited lower CAC. These compounds exhibited features similar to those described for molecules with the same content of nucleobases and amino acids in terms of fluorescence emission. The secondary structure seemed to be dictated for the longer sequences by the interaction between phenylalanines; CD spectra were in fact dominated by the positive signal around 220 nm. The results of the Thioflavin T assay suggested the formation of amyloid-like structures. The morphology of the aggregates was globular for all compounds, except for (cFgF)_2_, that produces superimposed thin plates.

Conjugates of hydrophobic peptides to PNA were employed to obtain hydrogel to be used as CEST-MRI agents [54]. The hexapeptide LVAGK was conjugated at the N-terminus to PNA nucleobases; all conjugates aggregated upon the formation of beta-sheet structures, as demonstrated by CD and fluorescence spectroscopy. Only the peptide containing cytosine was able to produce a hydrogel; interestingly, the mixing of peptides conjugated to c and g nucleobases in a 1:1 stoichiometric ratio resulted in improving the mechanical properties of the hydrogel as a consequence of Watson–Crick hydrogen bonding. The hydrogel exhibited a detectable CEST-MRI contrast and good biocompatibility, thus representing a valid alternative to Gd(III) complexes.

#### 2.4.3. Amphipathic Peptide Conjugates

Amphipathic peptides composed of hydrophobic and charged amino acids are well known for their ability to form hydrogels. The conjugation of short and long PNA sequences was reported by several research groups; it is demonstrated that the presence of PNAs improves the mechanical properties of the hydrogel.

In 2017, Di Maio et al. reported the conjugation of the (FKFE)_2_NH_2_ peptide to a 10-mer PNA to give PPH1 (Ac-PNA-PEG-peptide) and PPH2 (Ac-peptide-PEG-PNA-K) [26] (Figure 1C). PNA sequences in PPH1 and PPH2 were different and non-complementary. A mixture of 500 µM (FKFE)_2_NH_2_ peptide plus PPH1 and PPH2 in water produced fibrils, tapes, and helical ribbons. The peptide/PPH1/PPH2 ratio was 5:1:1. CD showed the presence of beta-sheets. Mixtures formed soft hydrogels. The addition of a DNA sequence complementary to both PNAs of PPH1 and PPH2 in an approximately 1:1 molar ratio with PNAs resulted in gels with a doubled storage modulus and an unmodified loss modulus. The rigidification of the gel and enhancement of the crosslinked entangled state were suggested by rheological measurements but were not visible in the TEM images.

A similar peptide motif with a shorter sequence (FEFK) conjugated to PNA dimers (ac or tg) was described by Hoschtettler et al. [55]. Only the peptide conjugated to the PNA tg was able to form a soft hydrogel at a concentration of 12 mM at pH 7.4. By contrast, by mixing equimolar amounts of ac-FEFK with tg-FEFK, a hydrogel could be obtained. The presence of complementary nucleobases resulted in a synergistic effect in the coassembly process, which translated into a very high value of G’. These gels displayed thixotropic behavior. Analysis by TEM revealed the presence of entangled fibers, organized in such a way as to form alveoli that can host the solvent. NMR analysis of the mixture revealed the presence of signals that could be attributed to the imido proton of thymine and the imino proton of guanine, suggesting the presence of hydrogen bonds between complementary bases.

The conjugation of a single PNA monomer to a hydrogel-forming peptide was reported by Giraud et al. [56]. The peptide (FDFDFKFK) displayed alternated charged and hydrophobic amino acids and formed translucent hydrogel at pH 2–3 and in the pH range between 7 and 12 [57]. In the conjugates, the phenylalanine residue at the N-terminus of the peptide was replaced by a PNA monomer. Conjugates of the peptide to PNA monomers formed a hydrogel in all pH ranges between 2 and 11 at a concentration of 15 mM in 6 h. While the conjugation of pyrimidines (c and t) did not affect the mechanical properties of the hydrogel, the conjugation of purines (a and g) led to a dramatic increase in the G’ module of the hydrogel. All gels showed thixotropic behavior. TEM images revealed that purine conjugates produced long, entangled fibers, while pyrimidine conjugates formed a mixture of thin fibers and spherical nanostructures. The different mechanical properties of the purine/pyrimidine hydrogels were consistent with the morphology of the self-assembled molecules.

Multicomponent hydrogels, formed upon mixing the PNA–peptide with PNA complementary bases, were also investigated. [58]. Unlike the monocomponent hydrogels, these do not show thixotropic behavior, but they display excellent thermoreversibility and very high stiffness. 

Amphiphile composed of the RGD-FF peptide conjugated at the N-terminus to a PNA monomer (t or a) and at the C-terminus to a fluorophore (rhodamine (Rh) or naphtalimide (Nph)) were used to produce crosslinked fibers [59]. In these molecules, the fluorophores, together with the dipeptide FF, triggered self-assembly, and the nucleobases stabilized the interactions between the assembling units thanks to hydrogen bond formation. The RGD peptide promoted the interactions of the assembled system with cancer cells expressing integrins on the cell surface. The network formed by Nph-a and Rh-t was able to recognize and encapsulate cancer cells displaying on the surface the integrin receptor, ultimately affecting cell viability. Interestingly, the assembly formed by Nph-a produced fibers while those produced with Rh-t were spherical.

The use of a PNA duplex to crosslink fibers formed upon the assembly of peptides was recently reported [60]. The peptide contains 20 amino acids and belongs to a family of beta-hairpin peptides, known to form hydrogels. Two PNA–peptide conjugates were obtained by ligating two 10-mer complementary PNA sequences to the peptide; the addition of 2% in mol of the PNA duplex to the peptide resulted in a 100% increase in the mechanical stiffness of the hydrogel. The duplex modulated the elastic component of the gel, facilitating the shear-thin recovery behavior. The morphology of the fibers, formed in the presence of the PNA duplex as observed by TEM, was similar to that observed for the peptide. Interestingly, two drugs (BAY and Amonafide) could be incorporated into the gels and released.

### 2.5. Assembly of Gamma-PNA

The introduction of a chiral center on the PNA backbone resulted in a defined organization of PNA oligomers and increased affinity of and specificity of binding toward complementary oligonucleotides [61]. The nature of the side chain as well as the number of modified monomers affected the behavior of the oligomers. PNAs modified with a diethylenglycol unit (miniPEG) in the gamma position of the backbone were designed to form nanofibers, exploiting the single-stranded tile technology [62]. Assembly occurred in organic solvents, namely in DMSO/water mixtures. The use of monomers modified on the backbone resulted in the enhancement of cooperativity in the nanostructure formation. Nine oligomers with a length of 12 bases, containing three modified PNA monomers (25% of the total), were employed to obtain three interwoven double helices. In the presence of SDS, nanofibers with a tight width distribution were obtained. When mixtures of DNA and gamma-PNA were used to form fibers, the morphology was affected by the surface and solvent exposure of DNA.

PNA oligomers displaying the same nucleobase sequence tested for miniPEG PNAs described earlier [62], modified with a serine side chain, formed nanofibers if the percentage of modified bases was between 67 and 100% (Figure 1A) [23]. In addition, such modified PNAs can form fibers by exploiting DNA templates.

Amphiphilic PNA oligomers, containing separated blocks of hydrophobic and hydrophilic gamma-PNA, named bilingual PNA, were employed to form stimuli-responsive spherical assemblies [63]. Initial investigations were carried out on oligomers in which the hydrophobic PNA domain contained two monomers with the alanine side chain, while the hydrophilic domain contained two monomers with the lysine side chain. These PNAs assembled in aqueous conditions to form micelles with a hydrophobic core and expose the hydrophilic moieties to the solvent. Disassembly occurred upon the addition of an oligonucleotide sequence complementary to the PNA. In this construct, the assembly process was harnessed by the peptide code, while the disassembly was achieved by exploiting the nucleotide recognition code. It was also possible, with a different combination of PNA sequences, to enable stimuli-responsive assembly using the nucleic acid code [64].

The properties of the assemblies can be tuned by changing the side chain and the spacing between the modified and unmodified monomers [32]. For example, the combination of PNAs with a hydrophilic moiety containing the side chain of glutamic acid and a hydrophobic moiety containing the side chain of leucine resulted in oligomers with a lower tendency to assemble, as demonstrated by the higher CMC, forming micelles of similar dimensions to the Ala/Lys side chain combination (Figure 3B) [32]. Decreasing the length of PNA oligomers affected the dimension of the micelle. 

Amphiphile PNAs were obtained by conjugating lauric acid (C12) to an 8-mer PNA fully modified with miniPEG in the gamma position of the backbone (Figure 3C) [33]. The PNA sequence was complementary to the seed region of oncomiR-155. By exploiting ethanol injection-based protocols, nanoassemblies with an ellipsoid morphology were obtained. These nanoassemblies were efficiently uptaken by U2932 lymphoma cells, mainly by macropinocytosis. The knockdown of miR-155, essential for lymphoma cell proliferation, was observed along with a decrease in cell viability. This is the first example of a functional gamma-PNA.

Spin-selective photoelectron transmission was observed in self-assembled monolayers formed by a 20-mer gamma-Ser PNA duplex [65]. The ability to transmit electrons with a specific spin orientation is typical of monolayers of chiral molecules, with chiral molecules as spin filters, and these molecules find application in enantioselective catalysis. These compounds can be used to control the selectivity of electrocatalytic reactions. A relationship between the helical sense and the direction of the transmitted electron was detected; finally, the spin polarization per length was higher in gamma-PNA duplexes than in dsDNA. 

## 3. Conclusions and Future Perspectives 

The chemical structure of PNAs and their ease of synthesis and modification render PNAs ideal and versatile substrates for the construction of new supramolecular systems. Studies reported so far demonstrate that short PNA sequences with the standard aminoethylglycine backbone can self-assemble only at high concentrations, but the addition of crosslinkers or modifications aimed at increasing the hydrophobicity of PNA oligomers strongly promotes their aggregation. The morphology of the aggregates is dependent on the chemical structure of the hydrophobic tail; as an example, when alkyl chains are attached to the PNA sequences, micelle-like structures can be obtained. When aromatic peptides are conjugated to the PNA, fibrils are formed if the interactions between the peptides are strong enough, as in the case of tetraphenylalanine. In other cases, when the interaction between the nucleobases and the peptide moiety both contribute to holding the supramolecular structure, the morphology of the aggregates is usually not very well defined. Interestingly, the onset of fluorescence was observed upon the aggregation of PNAs.

The combination of PNAs with amphipathic peptides was widely investigated and demonstrated to be a robust strategy for obtaining hydrogels. The recognition of PNA complementary bases is often exploited to crosslink peptide fibers, thus increasing the stiffness of the gels. 

Applications explored so far for PNA supramolecular structures range from the development of delivery systems for nucleic acids (exploiting the Watson–Crick recognition code) to methods for obtaining new biomaterials for biological applications, and new catalytic systems exploiting the ability of the aggregates to transfer energy and electrons. Studies on the relationship between the chemical structure of the aggregating building blocks and the morphology and physical properties of the assembled systems are needed; these will help us understand the rules at the base of self-assembling to achieve a rational design of PNA-based (bio)materials.

## Figures and Tables

**Table 1:** Self-assembling molecules containing PNAs.

Sequence ^1^	Morphology ^2^	Application ^3^
Biotin-tagagtt-Lys-NH_2_	Fiber (after DNA hybridization)	Hydrogel
Methacryloyl-Gly_2_-agtgaccg-OH/HPMA copolymer Methacryloyl-Gly_2_-accaggcg-OH/HPMA copolymer Methacryloyl-Gly_2_-ttctcttcttc-OH/HPMA copolymer Methacryloyl-Gly_2_-cttcttctctt-OH/HPMA copolymer	Porous structure (after DNA hybridization)	Hydrogel
Glu-tgagcttgtatagtcg-Glu * Glu-caagctcacgactata-Glu *	Film and hollow capsule	Delivery
aaaaaaa *	Fiber (after cyanuric acid binding)	
Fmoc-g(Bhoc)-OH	Microsphere	Photonic crystals Organic solvents detection
Fmoc-g(Bhoc)-TPP Fmoc-g(Bhoc)-BDP	Nanosphere Spherical or flake-shaped aggregate	Catalysis photodynamic therapy
Fmoc-g-aminopentanoic acid	Sphere or nanoribbon	Energy transfer processes
gc-NH_2_c g-NH_2_ gg-NH_2_	Crystal	Optoelectronic
Fmoc-gc-NH_2_	Sphere	Fluorescence
C_12_-gg-NH_2_	Sphere	n.r.
C_12_-agtgatctac-(Glu)_4_-NH_2_	Micelle	n.r.
C_12_-tttccg-(Lys)_2_-NH_2_	Micelle	n.r.
C_12_-tagacg-(Glu)_2_-NH_2_	Ellipsoidal micelle	Separation of DNA oligomers
C_12_-ctgactga-(Glu)_4_-NH_2_	Spherical micelle	n.r.
(C_14_)_2_-(AEEA)_2_-agtgatctac-(Glu)_4_-NH_2_	n.r.	Liposomes (mixture with cholesterol and DSPC) for DNA delivery
(C_14_)_2_-agtgatctac-(Glu)_4_-NH_2_	n.r.	Liposomes (mixture with cholesterol and DSPC) for DNA delivery
(C_14_)_2_-tttccg-(Lys)_2_-NH_2_	n.r.	Liposomes (mixture with cholesterol and DSPC) for DNA delivery
LysLys(Lys(Lys-t_7_))(Gly)_3_(Ala)_3_Lys(palmitoyl)-NH_2_	Fiber	Hydrogels
Boc-(Phe)_2_-tz-A^N(Boc)^_2_-aeg-OEthyl Boc-(Phe)_2_-tz-G^N(Boc)^_2_-aeg-OEthyl	Hollow nanoparticle Hollow nanoparticle	Encapsulation Supercapacitors
a-(Phe)_2_-OH or g-(Phe)_2_-OH aa-(Phe)_2_-OH or gg-(Phe)_2_-OH c-(Phe)_2_-OH or cc-(Phe)_2_-OH t-(Phe)_2_-OH or tt-(Phe)_2_-OH	Amorphous conglomerate Fibrous nanostructure Entwined fiber Entwined fiber	n.r.
gc−(Phe)_2_ gc−(Phe)_2_−NH_2_ (Phe)_2_−gc (Phe)_2_−gc−NH_2_	Spherical aggregate Spherical aggregate Spherical aggregate Spherical aggregate	n.r.
(Trp)_2_−gc−NH_2_ (Trp)_2_−at−NH_2_	Spherical aggregate/ intertwined fiber Spherical aggregate	n.r.
(Phe)_4_−gc−NH_2_ (Phe)_4_−at−NH_2_	Vesicle like Twisted fiber	n.r.
c-Phe-g-Phe-NH_2_ (c-Phe-g-Phe)_2_-NH_2_ t-Phe-a-Phe-NH_2_ (t-Phe-a-Phe)_2_-NH_2_	Globular aggregate Thin plates Globular aggregates Globular aggregates	n.r.
c-LeuValAlaGlyLys-NH_2_	n.r.	Hydrogel for CEST-MRI
Ac-ttctctctga-PEG-(PheLysPheGlu)_2_-NH_2_ Ac-(PheLysPheGlu)_2_-PEG-tttctaatgt-Lys-NH_2_	Fibrils	Hydrogels
Ac-ac-FEFK-NH_2_ Ac-tg-FEFK-NH_2_	Entangled fibers	Hydrogel
a-(PheAsp)_2_(PheLys)_2_-OH g-(PheAsp)_2_(PheLys)_2_-OH t-(PheAsp)_2_(PheLys)_2_-OH c-(PheAsp)_2_(PheLys)_2_-OH	Entangled fibers Entangled fibers Thin fibers/spherical nanostructures Thin fibers/spherical nanostructures	Hydrogel Hydrogel Hydrogel Hydrogel
t-ArgGlyAspPhePheLys(rhodamine)-NH_2_ a-ArgGlyAspPhePheLys(naphthalimide)-NH_2_	Crosslinked spherical fibers Crosslinked fibers	Encapsulation cancer cells
Ac-VLTKVKTKV^D^PPTKVQVKVFV-(PEG_10_)_2_-tgttacgact-NH_2_ Ac-VLTKVKTKV^D^PPTKVQVKVFV-(PEG_10_)_2_-agtcgtaaca-NH_2_	Fiber (after duplex assembly)	hydrogel
a^γmp^ata^γmp^gcgt^γmp^tcac-NH_2_ g^γmp^cta^γmp^ttga^γmp^gtaa-NH_2_ g^γmp^aca^γmp^tctt^γmp^actc-NH_2_ c^γmp^tgg^γmp^cgtg^γmp^cgga-NH_2_ c^γmp^gcc^γmp^agcc^γmp^ctcg-NH_2_ biotin-g^γmp^tga^γmp^accg^γmp^aggg-NH_2_ a^γmp^gtt^γmp^ttga^γmp^tgtc-NH_2_ Cy3-a^γmp^aaa^γmp^ctac^γmp^agaa-NH_2_ t^γmp^ccg^γmp^catt^γmp^ctgt-NH_2_	Fiber (after combination of all nine single strand PNAs)	
(aatagcgttcac)^γSer^-NH_2_ (gctattgagtaa)^γSer^-NH_2_ (gacatcttactc)^γSer^-NH_2_ (ctggcgtgcgga)^γSer^-NH_2_ (cgccagccctcg)^γSer^-NH_2_ biotin-DEG-(gtgaaccgaggg)^γSer^-NH_2_ (agttttgatgtc)^γSer^-NH_2_ TAMRA-DEG-(aaaactacagaa)^γSer^-NH_2_ (tccgcattctgt)^γSer^-NH_2_	Nanofiber (after a combination of all nine single-strand PNAs)	
t^γLys^caa^γLys^catc^γAla^agt^γAla^cD-NH_2_ t^γLys^caa^γLys^catc^γLeu^agt^γLeu^cD-NH_2_ Dt^γAla^caa^γAla^catc^γGlu^agt^γGlu^c-Gly-NH_2_ t^γLys^caa^γLys^cat^γAla^ca^γAla^gD-NH_2_ t^γLys^ca^γLys^ac^γAla^at^γAla^cD-NH_2_	Spherical assembly (micelles)	n.r.
(C_12_)-PEG_3_-agcattaa-Lys-PEG_3_-TAMRA (C_12_)-PEG_3_-(agcattaa)^γmp^-Lys-PEG_3_-TAMRA	Ellipsoid nanostructures	Anticancer
cgtacaaacttagacaccag-Lys_3_-NH_2_ Lys-ctggtgtcta-NH_2_ + Ac-agtttgtacg-(CH_2_)_3_SH ctgg^γSer^tgt^γSer^cta^γSer^-Lys-NH_2_+ Ac-agtttgtacg-(CH_2_)_3_SH	Monolayer (in duplex form with complementary PNA strand)	Electrocatalytic reactions

^1^ * = the C-terminal end is not specified; HPMA = N-(2-hydroxypropyl)methacrylamide; Bhoc = benzytriloxycarbonyl; TPP = 5-(4-aminophenyl)-10,15,20-triphenyl porphyrin; BDP= 10-(4-aminophenyl)-5,5-difluoro-1,3,7,9-tetramethyl-5H-dipyrrolo [1,2-c:2′,1′-f][1,3,2]diazaborinin-4-ium-5-uide; AEEA = 2-aminoethoxy-2-ethoxy acetic acid; tz = triazole; aeg = aminoethylglycine; PEG = 8-amino-3,6-dioxaoctanoic acid; γmp = miniPEG-gamma-PNA; γSer = Ser-gamma-PNA; γLys = Lys-gamma-PNA; γAla = Ala-gamma-PNA; γLeu = Leu-gamma-PNA; γGlu = Glu-gamma-PNA; D = 4-dimethylamino-naphthalimide PNA monomer; TAMRA= 5-Carboxytetramethylrhodamine; PEG3= 11-amino-3,6,9-trioxaundecanoic acid linker. ^2^ DSPC = distearoylphosphatidylcholine. ^3^ n.r. = not reported; CEST = chemical exchange saturation transfer; MRI = magnetic resonance imaging.
